# Supplementary material for: The Ameliorative Effects of Saikosaponin in Thioacetamide-Induced Liver Injury and Non-Alcoholic Fatty Liver Disease in Mice
Source: Int J Mol Sci. 2021 Oct 21;22(21):11383. doi: 10.3390/ijms222111383 (PMC8583725; doi:10.3390/ijms222111383)
Supplement: Supplementary file 1 [file ijms-22-11383-s001.zip › ijms-1414096-supplementary.pdf]

Supplementary Materials

Figure S1: Changes in liver histology by Sirius Red staining (magnification 200×). Figure S2: Changes in Desmet–Scheuer score of liver inflammation in the TAA and SSd groups over 8 weeks. All data are reported as means ± SD (n = 8). ** $p < 0.01$. The severity of inflammation was graded using the Desmet–Scheuer classification (none, 0; minimal, 1; mild, 2; moderate, 3; and severe, 4) [116]. Figure S3: Changes in (a) IHC staining (magnification: 200×) and (b) hepatic IL-1β levels in the normal, TAA, and SSd groups. a–cData with different letters above the columns were significantly different on one-way ANOVA, and Duncan's test at $p < 0.05$ was used to compare the means of the three groups. Figure S4: Impact of SSd on (a) glucose tolerance score. (b) AUC at 120 min after glucose injection. Data are in the form of mean ± SD (n = 8). * $p < 0.05$; ** $p < 0.01$. Figure S5: (a) Oil-Red-O-stained sections of the liver (magnification 200×); (b) Quantitative analysis of hepatic oil droplets. Figure S6: UCP1 mRNA expression measured in brown adipose tissue from the control and SSd groups (all fed an HFD). *** $p < 0.001$. Figure S7: Changes in serum ALT and AST levels in the TAA-induced liver injury mice and SSd (1 mg/kg)-treated mice with TAA-induced liver injury over a period of 8 weeks. By employing primary antibodies against IL-1β (Merck, Billerica, MA, USA), we also executed further IHC staining for IL-1β in livers. We applied BioTnA's (Kaohsiung, Taiwan) TAlink mouse/rabbit polymer detection system to perform our protein expression measurement through IHC.

116 Eurich, D.; Boas-Knoop, S.; Ruehl, M., Schulz, M.; Carrillo, E.D.; Berg, T.; Neuhaus, R.; Neuhaus, P.; Neumann, U.P.; Bahra, M. Relationship between the interleukin-28b gene polymorphism and the histological severity of hepatitis C virus-induced graft inflammation and the response to antiviral therapy after liver transplantation. *Liver Transpl.* **2011**, *17*, 289–298.

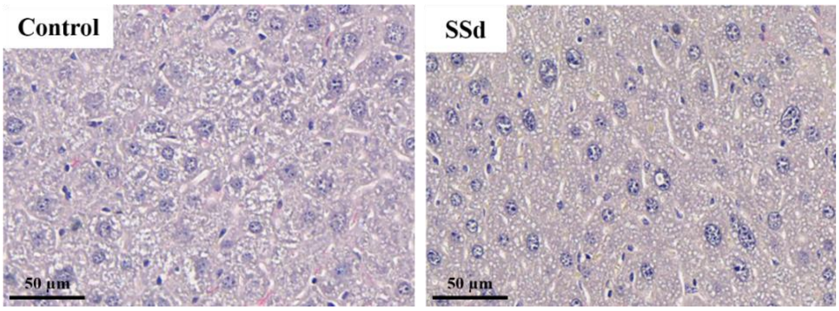

Figure S1. Changes in liver histology by sirius red staining (magnification 200×).

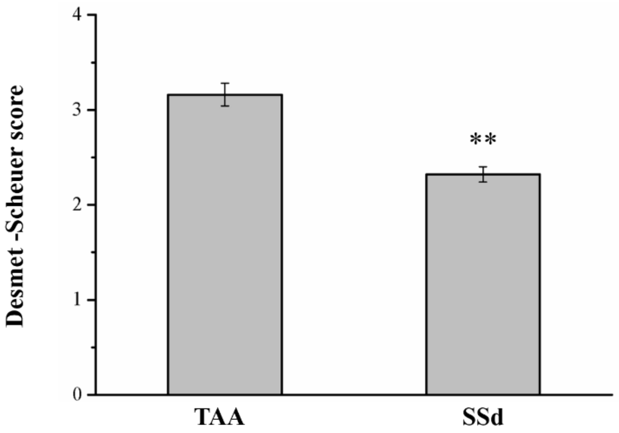

Figure S2. Changes in Desmet–Scheuer score of liver inflammation in the TAA and SSd groups over 8 weeks. All data are reported as means ± SDs (n = 8). ***p* < 0.01.

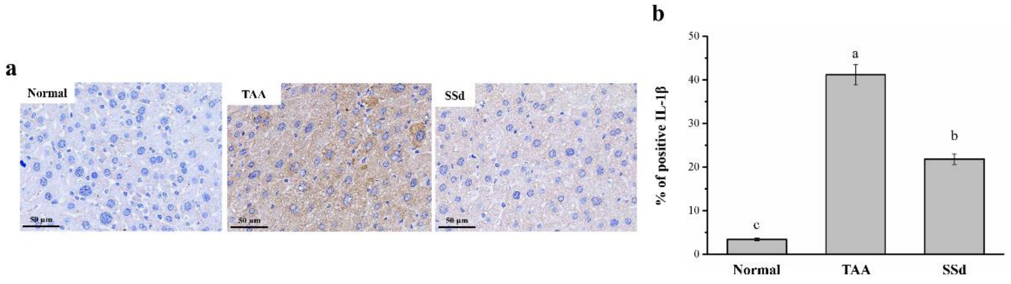

Figure S3. Changes in (a) IHC staining (magnification: 200×) and (b) hepatic IL-1β levels in the normal, TAA, and SSd groups. a–cData with different letters above the columns were significantly different on one-way ANOVA, and Duncan's test at $p < 0.05$ was used to compared the means of the three groups.

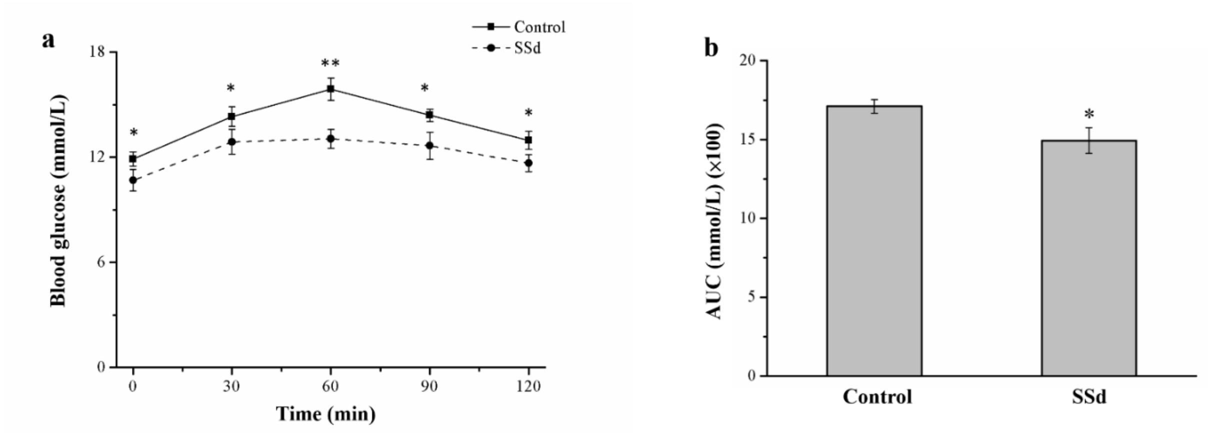

Figure S4. Impact of SSd on (a) glucose tolerance score. (b) AUC at 120 min after glucose injection. Data are in the form of mean ± SD (n = 8). **p* < 0.05; ***p* < 0.01.

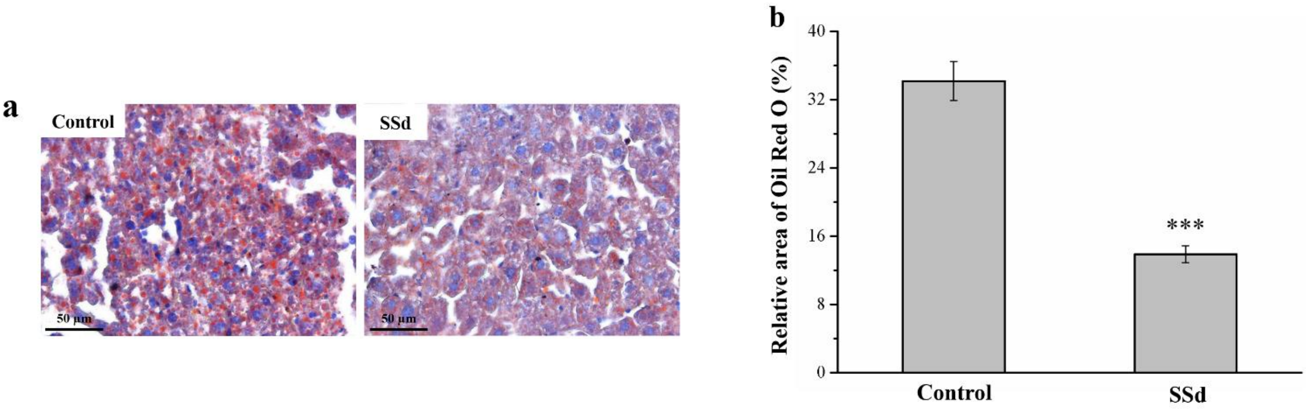

Figure S5. (a) Oil-Red-O-stained sections of the liver (magnification 200×); (b) Quantitative analysis of hepatic oil droplets.

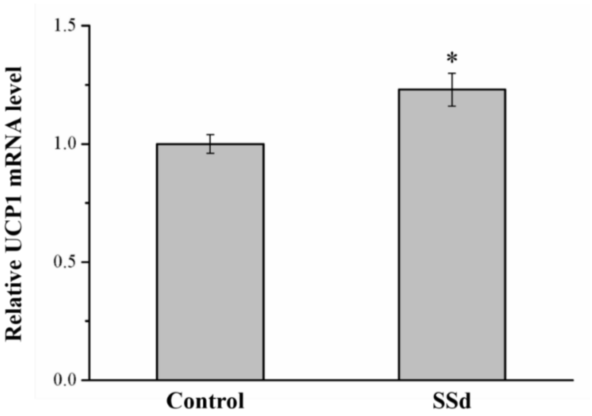

Figure S6. UCP1 mRNA expression measured in brown adipose tissue from the control and SSd groups (all fed an HFD). ***$p < 0.05$.

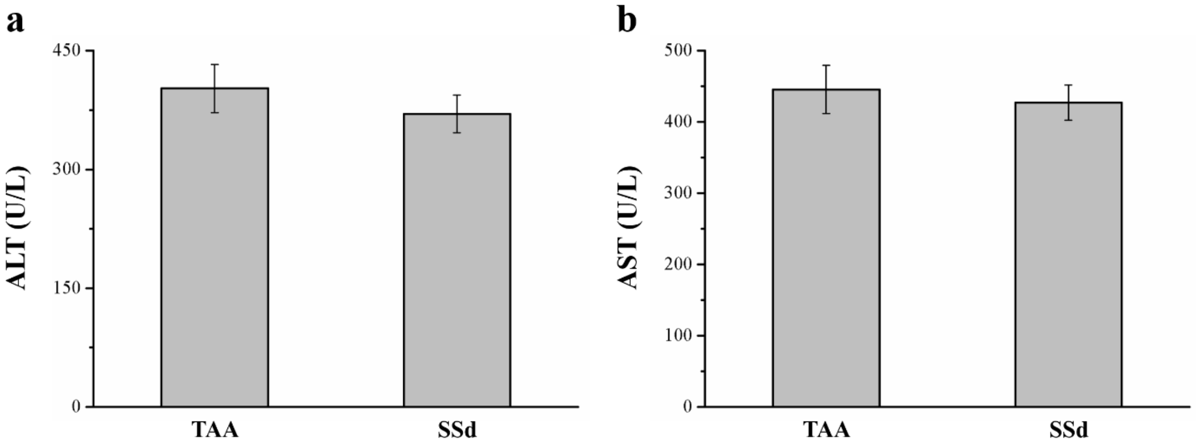

Figure S7. Changes in serum ALT and AST levels in the TAA-induced liver injury mice and SSd (1 mg/kg)-treated mice with TAA-induced liver injury over a period of 8 weeks.
